# Optimal futility stopping boundaries for binary endpoints

**DOI:** 10.1186/s12874-024-02190-w

**Published:** 2024-03-28

**Authors:** Michaela Maria Freitag, Xieran Li, Geraldine Rauch

**Affiliations:** 1 Charité - Universitätsmedizin Berlin, corporate member of Freie Universität Berlin, Humboldt-Universität zu Berlin, Institute of Biometry and Clinical Epidemiology, Charitéplatz 1, 10117 Berlin, Germany; 2 Berlin Institute of Health (BIH), Anna-Louisa-Karsch-Str. 2, 10178 Berlin, Germany; 3 Technische Universität Berlin, Straße des 17. Juni 135, 10623 Berlin, Germany; 4 medac GmbH, Theaterstraße 6, 22880 Wedel, Germany

**Keywords:** Single-arm phase II trial, Futility stop, Group sequential design, Binary endpoint

## Abstract

**Background:**

Group sequential designs incorporating the option to stop for futility at the time point of an interim analysis can save time and resources. Thereby, the choice of the futility boundary importantly impacts the design’s resulting performance characteristics, including the power and probability to correctly or wrongly stop for futility. Several authors contributed to the topic of selecting good futility boundaries. For binary endpoints, Simon’s designs (Control Clin Trials 10:1–10, 1989) are commonly used two-stage designs for single-arm phase II studies incorporating futility stopping. However, Simon’s optimal design frequently yields an undesirably high probability of falsely declaring futility after the first stage, and in Simon’s minimax design often a high proportion of the planned sample size is already evaluated at the interim analysis leaving only limited benefit in case of an early stop.

**Methods:**

This work focuses on the optimality criteria introduced by Schüler et al. (BMC Med Res Methodol 17:119, 2017) and extends their approach to binary endpoints in single-arm phase II studies. An algorithm for deriving optimized futility boundaries is introduced, and the performance of study designs implementing this concept of optimal futility boundaries is compared to the common Simon’s minimax and optimal designs, as well as modified versions of these designs by Kim et al. (Oncotarget 10:4255–61, 2019).

**Results:**

The introduced optimized futility boundaries aim to maximize the probability of correctly stopping for futility in case of small or opposite effects while also setting constraints on the time point of the interim analysis, the power loss, and the probability of stopping the study wrongly, i.e. stopping the study even though the treatment effect shows promise. Overall, the operating characteristics, such as maximum sample size and expected sample size, are comparable to those of the classical and modified Simon’s designs and sometimes better. Unlike Simon’s designs, which have binding stopping rules, the optimized futility boundaries proposed here are not adjusted to exhaust the full targeted nominal significance level and are thus still valid for non-binding applications.

**Conclusions:**

The choice of the futility boundary and the time point of the interim analysis have a major impact on the properties of the study design. Therefore, they should be thoroughly investigated at the planning stage. The introduced method of selecting optimal futility boundaries provides a more flexible alternative to Simon’s designs with non-binding stopping rules. The probability of wrongly stopping for futility is minimized and the optimized futility boundaries don’t exhibit the unfavorable properties of an undesirably high probability of falsely declaring futility or a high proportion of the planned sample evaluated at the interim time point.

**Supplementary Information:**

The online version contains supplementary material available at 10.1186/s12874-024-02190-w.

## Background

Single-arm trials with binary endpoints are commonly used in oncological phase II studies. For example in the field of oncology, some trials rely on response rate as short-term endpoints to scan for an early indication of anti-tumor activity as a go or no-go decision for conducting further large phase III trials. Adaptive trial designs offer the benefit of possibly faster decisions due to the opportunity for early termination. The option to stop for futility is an important feature in adaptive trials, especially in phase II trials when screening for possible treatments plays a much greater role than proof of efficacy. It is important that the decision on which treatments to continue investigating is reliable and made as soon as possible so that the number of patients treated with potentially ineffective treatments is minimized. Therefore, several authors investigated the theory behind optimal futility boundaries in general [1–5] and the time point of the interim analysis [6, 7]. There are many different two-stage designs for single-arm phase II clinical trials with binary endpoints. The first one was introduced by Gehan [8] in 1961. Simon’s two-stage designs [9] from 1989 were commonly used in practice and numerous adaptations of this design have been investigated. The most well-known are the admissible two-stage designs by Jung et al. [10] which present a trade-off between Simon’s optimal and minimax design. However, the balance between the sample sizes of the first and second stages is rarely taken into account, even though it is important to achieve a good ethical benefit: If the interim analysis for futility is carried out with too little data, correct decision-making will be difficult and if the interim analysis is conducted at a late time point a potential early stop provides hardly any benefit in patient saving, as e.g. Lawrence Gould points out [7]. Kim et al. [11] contributed to incorporating a reasonable time point for the interim analysis into Simon’s designs. They put an upper and a lower bound on the time point, among other constraints, and used the expected sample size under the null hypotheses and the total sample size as the optimization criteria for the modified versions of Simon’s optimal and minimax designs, respectively. In most of the previous works, the expected sample size is a key performance parameter for futility stopping boundaries. Although this is an important operational characteristic, it should not be used as the only performance measure. Some existing works [12, 13] investigate the sample size together with the power as a combined performance score. The overall power has to be controlled in clinical trials [14] to favor positive results when the null hypothesis should indeed be rejected. Therefore, the power loss introduced by futility stopping is also an important performance measure. Also, the probability of wrongly stopping for futility at the interim analysis should be low for not throwing the existing resources of conducting the trial in vain. Schüler’s and Li’s publications [15, 16] investigate futility stopping boundary based on both power loss and probability of wrongly stopping for futility while maximizing the probability of correctly stopping for futility. Schüler et al. (2017) discussed these optimality criteria for futility boundaries for the special case of trials with (multiple) time-to-event endpoints [15]. Their concept was adopted by Li et al. for continuous endpoints [16].

There are two types of futility stopping boundaries: binding and non-binding. When using a binding rule, the trial must be stopped when the corresponding criteria are fulfilled – regardless of external input and other factors. In exchange, the critical boundaries (i.e. the thresholds for the actual testing) can be increased to fully exhaust the targeted nominal significance level $\alpha$. This is because the option of stopping for futility reduces the probability of rejecting the null hypothesis, which also reduces the power [17]. This adjustment of the critical boundary could allow a smaller sample size needed to meet the desired values for $\beta$ and overall $\alpha$. However, if a binding stopping rule for futility is ignored and the study nevertheless continues to the second stage the overall type I error rate is inflated, as binding boundaries are derived with an assumption requiring early stops whenever the boundary is crossed. This is not the case with non-binding rules. There, no critical boundary can be adjusted and the study can continue e.g., if external information suggests that the criteria have been set too strictly or a secondary endpoint seems promising. Non-binding rules are more commonly applied in conducted clinical trials, as flexibility is preferred and stopping for futility often is a multidimensional decision not solely based on the interim results but also including the total evidence of the data like e.g. toxicity and also new external data can sometimes influence the decision in addition to the interim data generated by the trial in question. In our approach, we will not adjust the critical boundary to allow for this kind of flexibility and non-binding decisions. However, in absence of solid reasons adherence to the stopping rule is expected in order to protect patients from inefficacious regimens – even if non-binding futility boundaries are used.

The methods by Simon and Kim et al. adjust the critical boundaries incorporating the binding futility stopping rules so that overall the type I error will be controlled by the pre-specified targeted nominal significance level $\alpha$. This means the assumption that the binding rule is strictly followed is part of the design algorithm searching for the critical boundaries while controlling the type I error. By translating the critical boundaries to the p-value scale, this results in a threshold higher than the targeted nominal significance level $\alpha$, in case of non-adherence to the binding rule.

## Methods

After introducing some notations for binary endpoints, we extend Schüler’s definition of “optimal” futility boundaries to single-arm studies with a single binary endpoint. First, we introduce the analytic algorithm to derive these futility boundaries. Then the performance of study designs with an optimized futility boundary are compared to Simon’s minimax and optimal designs, as well as modified versions of these designs by Kim et al. [9, 11].

### The test problem

Consider a single-arm, two-stage design with a binary endpoint variable $Y_i = 1$ if the event occurs and $Y_i = 0$ if it does not, with $i = 1 \ldots n$ for a given sample size *n*. In the first stage, $n_1$ patients are evaluated, and in the second stage $n_2=n-n_1$ patients. For pre-specified null and alternative response proportions $p_0$ and $p_a$ with $p_0 < p_a$, the test problem reads as

1 $$\begin{aligned} H_0: p \le p_0 \ \text {versus} \ H_1: p > p_{0}. \end{aligned}$$

The value $p_0$ denotes the maximum response probability so that the treatment would be deemed unsuccessful and $p_a$ is the minimum response probability in order to warrant further studies. If for the true response probability it holds that $p \le p_0$ the probability to reject the null hypothesis should be below a pre-specified targeted nominal significance level $\alpha$. If it holds that $p \ge p_a$ this probability should be above the pre-specified power level $1-\beta$. However, rejecting the null hypothesis does not necessarily imply for the true response rate to be larger than $p_a$ which the study is powered for. Suppose $x_1$, $x_2$, and *x* are the number of responses from the first stage, the second stage, and the total responses from both stages combined, respectively. The trial will be stopped after the first stage if $x_1 \le r_1$ out of $n_1$ patients, where $r_1$ is the futility threshold. Otherwise, the trial proceeds to the second stage, and additional $n_2$ patients are recruited. If $x > r$ the new treatment is considered effective, otherwise the trial fails to reject the null hypothesis. Due to limited sample size in these designs, test statistics are based on the exact binomial test, evaluating the exact ratio of *r* and *n*. The rejection region is defined as

2 $$\begin{aligned} \Omega = \{ (x_1, x) : x_1> r_1\ \text {and}\ x>r \}. \end{aligned}$$

The corresponding probability is calculated as the sum of the conditional probability of $x_2$ given $x_1$ where *b*(*r*, *n*, *p*) denotes the binomial probability mass function and *B*(*r*, *n*, *p*) the cumulative binomial distribution function:

3 $$\begin{aligned}& \sum _{i=r_1+1}^{min(n_1,r)} P(x_1=i) P(x_2>r-i|x_1=i)\nonumber \\& = \sum _{i=r_1+1}^{min(n_1,r)} b(i,n_1,p) \cdot (1-B(r-i,n_2,p)). \end{aligned}$$

The required sample size *n* to achieve the desired targeted nominal significance level $\alpha$ and power $1-\beta$ is determined together with $r_1$ and *r*. The overall probability to reject $H_0$ after stage 2 can be controlled by the global level $\alpha$ as

4 $$\begin{aligned}& 1 - [B(r_1,n_1,p_0) \nonumber \\& + \sum _{i=r_1+1}^{min(n_1,r)}b(i,n_1,p_0)B(r-i,n_2,p_0)] \le \alpha . \end{aligned}$$

It should be noted that even though $\alpha$ and $\beta$ are set as desired targeted type I and type II error rates they are usually not reached exactly. They instead act as upper boundaries for the actual design specific error rates $\alpha ^d \le \alpha$, and $\beta ^d \le \beta$ as the test statistic is non-continuous.

### Simon’s and the modified Simon’s two-stage designs

For given values of $\alpha$, $\beta$, the upper bound for the sample size $n_{max}$ and given rates $p_0$ and $p_a$ Simon’s designs are characterized by four values $r_1$, $n_1$, *r*, and *n*. Within all possible tuples $(r_1, n_1, r, n)$ fulfilling the desired constraints for $\alpha$ (as in the Eq. 4), $\beta$ and $n_{max}$, Simon’s optimal design selects the one, that minimizes the expected sample size under the null hypothesis and Simon’s minimax design the one that minimizes the maximum sample size *n*. Kim’s modified minimax and optimal designs [11] put two additional constraints on the tuples - namely, a bound for the probability of wrongly stopping for futility and a range for the proportion of the sample size evaluated at the interim analysis. The same criteria to select the optimal tuple as in Simon’s designs are applied.

In the case of Simon’s optimal design, the probability of wrongly stopping for futility can be undesirably high. In the minimax design, often such a large proportion of the total number of cases is evaluated at interim that the benefit from the interim analysis is limited. The modified Simon’s optimal and minimax designs and the introduced optimized futility boundaries address those issues and keep the corresponding characteristics in pre-specified bounds.

### Optimality criteria for futility boundaries

Various criteria are quantifying the performance of adequate futility boundaries. One goal is to avoid the power loss caused by the option of an early futility stop becoming too large if the alternative hypothesis holds true. In this regard, the interim analysis should make as many correct decisions as possible. Thus, the probability of stopping correctly for futility should be as high as possible and the probability of stopping incorrectly as low as possible. Unfortunately, it is not possible to optimize all those performance measures at the same time. If the stopping boundary at interim is large, we will often stop correctly, but the power loss can be very high and the study may too often be stopped wrongly. On the other hand, if the boundary is weak (i.e. a small boundary value for the responses), the loss of power is small, but at the same time, it is also rare to stop correctly if there is only a very small effect or even an opposite effect. One approach is to find a balance between these characteristics. In this sense, admissible conditions for selecting futility boundaries were defined in the paper by Schüler et al. (2017) for time-to-event endpoints and adopted in a paper by Li et al. (2020) to continuous endpoints [15, 16]. The basic idea is to maximize the probability of correctly stopping for futility under the null hypothesis, i.e., to increase the probability of correctly identifying very small or opposite treatment effects in the interim analysis while keeping both the loss of global power under the alternative limited as well as the probability of incorrectly stopping for futility.

Some parameters are introduced for this concept: Let $Pow_{\text {loss}}<1-\beta$ denote the acceptable overall power loss compared to the power that would be reached in a trial with the same total sample size but without an interim analysis. This sample size refers to the smallest providing at least a power of $1-\beta$ in a one stage trial. Moreover, the probability of wrongly stopping for futility, i.e. the probability of early termination under $p_a$ PET($p_a$), should be limited by $\pi _{\text {wrong}}\in [0,1]$ given the underlying treatment effect is in fact at least the assumed minimally relevant effect $p_a$. The publications by Schüler et al. and Li et al. apply a different method of formalizing the stopping rule than the one we introduced for Simon’s designs [15, 16]. There the study is stopped for futility if the *p*-value at the time point of the interim analysis is $p_{interim}> \alpha _{0}$, where $\alpha _{0}$ is the futility boundary. The null hypothesis $H_0$ is rejected at the final analysis if the corresponding *p*-value is smaller or equal to the one-sided significance level $p_{total}\le \alpha _{}$. Both methods of formalizing the stopping rule can equivalently be converted into each other. Using the *p*-value notation for decision-making, a futility boundary fulfills the so-called **admissible conditions** [15, 16] if the following requirements are satisfied:

1. $$P_{p_a}(p_{interim} > \alpha _{0})$$ = PET($$p_a$$) $$\le \pi _{\text {wrong}}$$,
2. $$P_{p_a}\left( p_{interim} < \alpha _{0} \cap p_{total} \le \alpha )\right) \ge 1-\beta -Pow_{\text {loss}}$$.

Any futility boundary fulfilling the admissible conditions is denoted as $\alpha _{0,\text {ad}}$. For predefined values $Pow_{\text {loss}}$ and $\pi _{\text {wrong}}$ there exists a whole set of admissible futility boundaries fulfilling the above conditions. Within this set, the “optimal” futility boundary is selected as the one with the highest probability of correctly stopping for futility, i.e. the probability of early termination under $p_0$, given by PET($p_0$). Although there are more elegant mathematical approaches to define good futility boundaries, like optimization problems with constraints [3, 4] or maximizing utility functions [5], the advantage of Schüler’s approach is that this concept of “optimized” futility boundaries is simple and easy to communicate to applied researchers. The expression “optimized” refers to the investigated performance indicators and there is no claim to a universal best solution. In the publications of Schüler and Li, the interim analyses were conducted when half of the total sample size had been reached [15, 16]. Commonly a futility boundary for the *p*-value of 0.5 is implemented in clinical trials with these kinds of endpoints as it prompts to stop the trial whenever the treatment effect points in the wrong direction. For example, the software ADDPLAN sets this value as the default [18] and the R-package *rpact* uses this value for its illustrating examples of the use of futility boundaries [19]. Therefore, these publications use the boundary value 0.5 as a reference point even though there is no unique standard. The futility boundaries found by the introduced method were mostly smaller than 0.5 and provided in many cases better performance characteristics than the designs with the standard futility boundary of 0.5. However, the works of Schüler et al. and Li et al. [15, 16] refer to two-armed trials with time-to-event or continuous endpoints, and the performance for a single-arm trial with a binary endpoint was not yet evaluated.

### Application to the single-arm, binary case

We apply the general formulation of admissible conditions from Schüler and Li [15, 16] to the specific case of a binary endpoint in a single-arm trial. For this purpose, we rephrase the admissible conditions in terms of boundaries for the response rates instead of boundaries for *p*-values. Additionally, we describe the algorithm used to derive optimized boundaries.

First, the sample size (and also the critical value *r*) are selected such that a given effect can be detected with power $1-\beta$ and a one-sided targeted nominal significance level $\alpha$ in a study without an interim analysis. Next, $r_1$ and $n_1$ are selected by determining all admissible values for $n_1$ and $r_1$ and then maximizing PET($p_0$) over this set. In this process, the critical boundary is not adjusted. Thus, the calculated bounds allow for the flexibility of non-binding futility decisions. The constraints on the admissible designs are similar to those in Schüler’s approach, which also used constraints for power loss $Pow_{loss}$ and the probability of wrongly stopping for futility $\pi _{wrong}$. Here, in addition to $Pow_{loss}$ and $\pi _{wrong}$, the interim analysis is not necessarily performed at 50% of the total sample size. So an additional condition for the interim time point is added and bounds ($ratio_{lower}$ and $ratio_{upper}$) for the ratio of $n_1$ to *n* can be specified by the user. Within these admissible boundaries, the probability of correctly stopping for futility PET($p_0$) is maximized.

The applied admissible conditions for the selection of $r_1$ and $n_1$ are:

1. $$ratio_{lower} \le \frac{n_1}{n} \le ratio_{upper}$$,
2. $$B(r_1,n_1,p_a)$$ = PET($$p_a$$) $$\le \pi _{\text {wrong}}$$,
3. $$1- \Big (\sum _{i=r_1+1}^{min(n_1,r)} b(i,n_1,p_a) \cdot B(r-i,n_2,p_a)+ B(r_1, n_1, p_a)\Big ) \ge 1-\beta -Pow_{\text {loss}}$$.

## Results

We compare the optimized design with Simon’s designs and Kim’s modified Simon’s designs. Additionally to the here presented results, we provide as Supplementary material an R-program which calculates the optimized futility boundaries for arbitrary pre-specified design parameters, and tables with specific design characteristics for additional scenarios.

### Investigated scenarios and operating characteristics

We have selected six operating characteristics based on which we want to compare the introduced optimized design with Simon’s optimal and minimax designs and Kim’s modified versions of these designs. The selected operating characteristics are:

1. the proportion of patients evaluated at the interim analysis, i.e. $n_1/n$,
2. the probability of wrongly stopping for futility at the interim analysis, i.e. the probability of early termination under the alternative hypothesis PET($p_a$),
3. the probability of correctly stopping for futility at the interim analysis, i.e. the probability of early termination under the null hypothesis PET($p_0$),
4. the actual type I error when ignoring the futility boundary, i.e. $\alpha ^d_\text {no stop}$,
5. the actual power, i.e. $1-\beta ^d$,
6. the maximum sample size and the expected sample sizes under the null and alternative hypothesis, i.e. $n = n_{max}$, EN($p_0$), and EN($p_a$) respectively.

The proportion of patients evaluated at the interim analysis $n_1/n$ should neither be too high nor too low to benefit most from the interim analysis, as described earlier [7]. As bounds for the fraction of patients investigated at the time point of the interim analysis, we selected $ratio_{lower} = 1/3$ and $ratio_{upper} = 2/3$ for all investigated scenarios. The probability of wrongly stopping for futility PET($p_a$) should be as low as possible so that more data can be collected on promising treatments. The exact admissible value is subject to the user’s choice. We selected $\pi _{\text {wrong}} = 0.05$ as the admissible parameter for the investigation. The probability of correctly stopping for futility PET($p_0$) should be as high as possible so that a high proportion of correct decisions is made at the interim analysis and fewer resources are used for futile treatments. Since decisions in the industry are often not solely based on the number of responses when they are in the margin area and sometimes additional external data is investigated, it is interesting to examine the type I error when not adhering to the stopping rule $\alpha ^d_\text {no stop}$ and treating the boundaries as non-binding. It would be desirable if in this case, the actual type I error would still be below $\alpha = 0.05$, but it could be inflated if the critical boundary was adjusted upwards due to binding futility stopping rules. For binary endpoints, the targeted power and the actual power often don’t match and the actual power can be higher. The actual power is subject to the targeted power and the admissible power loss which we set for these investigations to $Pow_{\text {loss}} = 0.05$. The higher the power the better. The last investigated operating characteristics, maximum sample size and expected sample sizes under the null and alternative hypotheses, are measures for the cost of the trial. The lower the sample sizes the fewer resources are needed and the faster the trial can be conducted.

In the following, we investigate these characteristics for possible designs with an assumed effect of $p_a-p_0 = 0.15$ and error rates $\alpha = 0.05$ and $\beta =0.2$ using a step width of 0.05 for $p_0$.

### Performance comparison

**Fig. 1:**
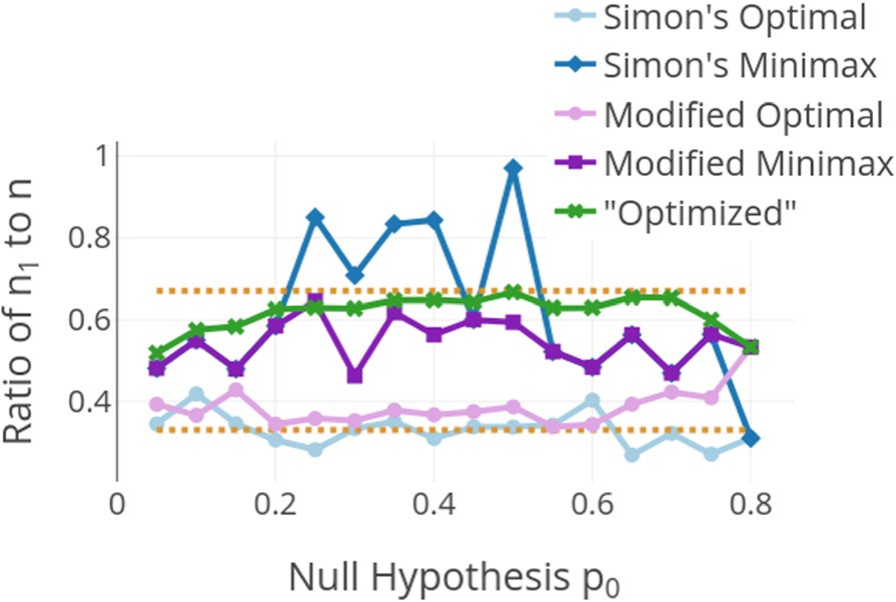
Ratio of $n_1$ to *n* for $p_a-p_0 = 0.15$, $\alpha = 0.05$ and $\beta = 0.2$. The dotted lines show the set boundaries for the proportion of $n_1$ to *n*, here 1/3 and 2/3

Figure 1 depicts the proportion of the sample size that is evaluated at the interim analysis. For the optimized design and Kim’s modified Simon’s designs a range for this ratio is pre-specified in the planning stage but the actual ratio is subject to the results of the algorithms. Simon’s original designs don’t set any constraints on this ratio. The x-axis gives the value for the null hypothesis $p_0$. The alternative hypothesis $p_a$ always corresponds to the value $p_0 + 0.15$. The exact study designs $(r_1, n_1, r, n)$ are not given in the figures but can be found in tables in the Supplementary material. The blue lines in the figures depict the classical Simon’s designs, the violet ones the modified Simon’s designs, and the green line the introduced new approach. For example, for the value 0.25 on the x-axis, we get the ratio of $n_1$ to *n* for $p_0=0.25$ and $p_a=0.4$ for the five designs. In Simon’s optimal design $(r_1, n_1, r, n)$ = (5, 20, 23, 71), a ratio of 0.28 of the total sample size is evaluated at the interim analysis, and in Simons’s minimax design $(r_1, n_1, r, n)$ = (16, 51, 20, 60) a ratio of 0.85. In Kim’s modified Simon’s optimal design $(r_1, n_1, r, n)$ = (6, 24, 22, 67) and minimax design $(r_1, n_1, r, n)$ = (10, 40, 21, 62) these values are 0.36 and 0.65 respectively. The introduced new design $(r_1, n_1, r, n)$ = (10, 39, 21, 62) evaluates a ratio of 0.63 at the interim analysis. The orange lines indicate the bounds $ratio_{lower}=1/3$ and $ratio_{upper}=2/3$ that were set in the planning stage. These bounds are an indicator of the performance as it is important for an efficient interim analysis that neither too few patients are included nor the proportion of the total data is too high. Simon’s designs often lie outside this range, as illustrated in Fig. 1 and the example above. The optimized design and Kim’s modified Simon’s designs are an improvement in this regard. The optimized designs often nearly exhaust the upper bound for the ratio whereas Kim’s modified optimal design tends towards the lower bound.

**Fig. 2:**
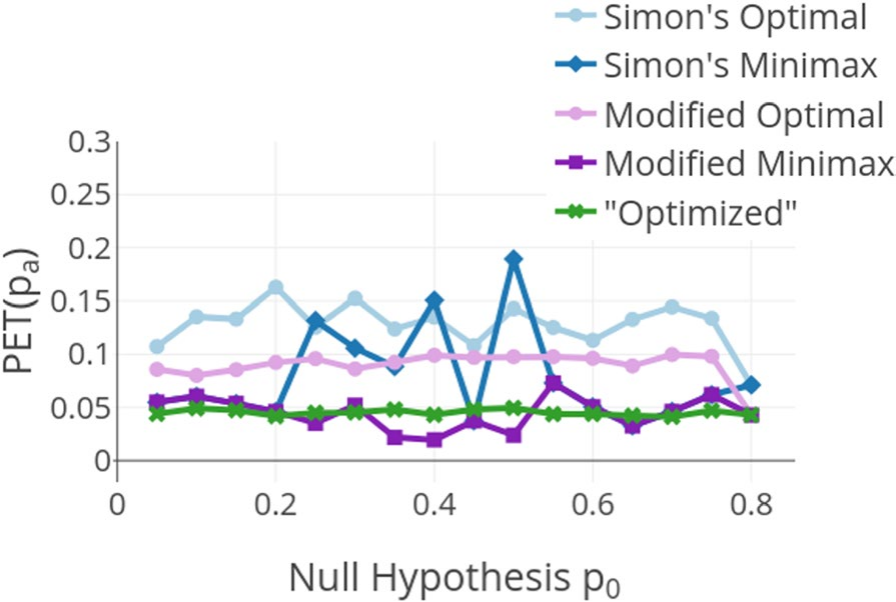
Probability of early termination under the alternative hypothesis $p_a$ for $p_a-p_0 = 0.15$, $\alpha = 0.05$ and $\beta = 0.2$. PET($p_a$) corresponds to the probability of wrongly stopping for futility at the interim analysis

The probability of wrongly stopping for futility PET($p_a$) which is the probability of early termination under the alternative hypothesis $H_a$ is always below the predefined threshold for the introduced optimized design (as depicted in Fig. 2). The figure shows that for the other designs this probability is much higher and goes as high as 18.9% for $p_0=0.5$ and $p_a=0.65$ in Simon’s minimax design. This means that they spend a much higher proportion of the allowed type II error rate after the first stage. Note however that it was a design choice for the modified Simon’s designs by Kim to control the probability of wrongly stopping for futility more loosely at 0.1. This input parameter (as well as the other input parameters) could easily be modified to accommodate the more strict 0.05 boundary but this would result in higher expected sample sizes as the ones depicted later in this Results section.

**Fig. 3:**
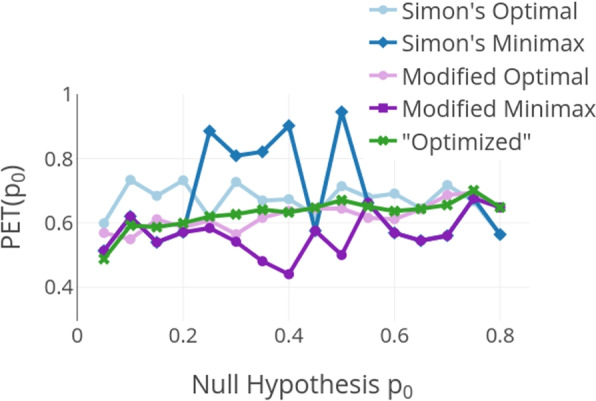
Probability of early termination under the null hypothesis $p_0$ for $p_a-p_0 = 0.15$, $\alpha = 0.05$, and $\beta = 0.2$. PET($p_0$) corresponds to the probability of correctly stopping for futility at the interim analysis

The probability of correctly stopping for futility PET($p_0$) in the introduced optimized design is mostly higher than in Kim’s modified Simon’s designs, see Fig. 3, even though the probability of wrongly stopping for futility is mostly lower. This makes the optimized design a better choice than the modified designs with regard to the proportion of correct decisions. The original Simon’s designs often have a higher probability of correctly stopping for futility than the modified designs and the introduced design. This is to be expected since the probability of wrongly stopping for futility is often also much higher and these values are correlated.

**Fig. 4:**
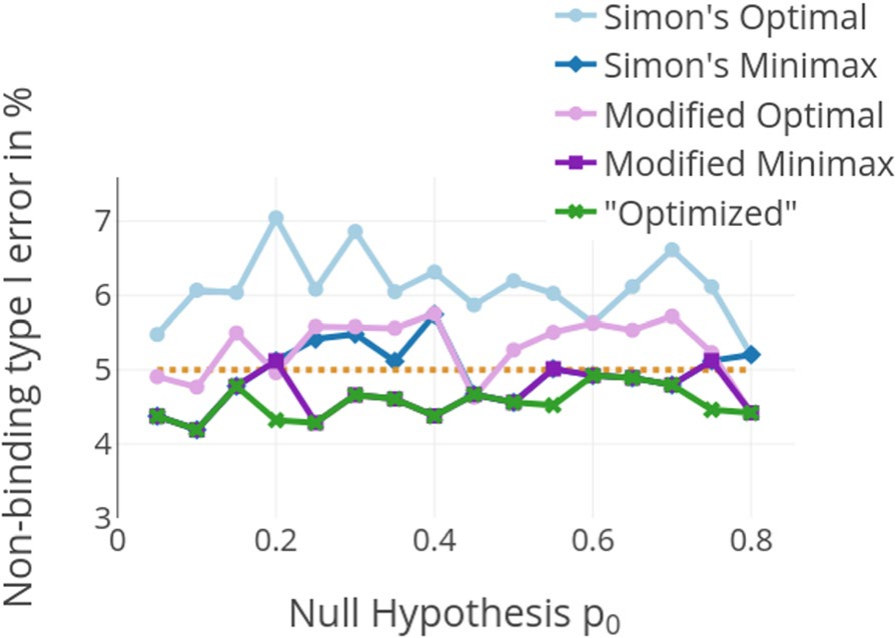
Type I error rate when not adhering to the stopping rule and always proceeding to the second stage $\alpha ^d_\text {no stop}$ for $p_a-p_0 = 0.15$, $\alpha = 0.05$ and $\beta = 0.2$. The introduced optimized design always keeps the type I error rate even if the stopping rules are not adhered to

All designs keep the prespecified type I error rate of 0.05 if the investigators follow the futility sopping rules. If they should for some reason choose to continue the trial nevertheless this is not always the case, as shown in Fig. 4. Simon’s designs and Kim’s modified designs do not consistently keep the type I error rate if the futility boundaries are treated as non-binding. Especially for Simon’s optimal design, the type I error can get very inflated up to about 7% for $p_0=0.2$ and $p_a=0.35$. This is because those designs apply binding futility stopping rules with adjusted critical boundaries. In contrast, by construction, the introduced method to derive optimized futility boundaries keeps the type I error under the predefined targeted nominal significance level of 0.05 even if the stopping rules are not adhered to.

**Fig. 5:**
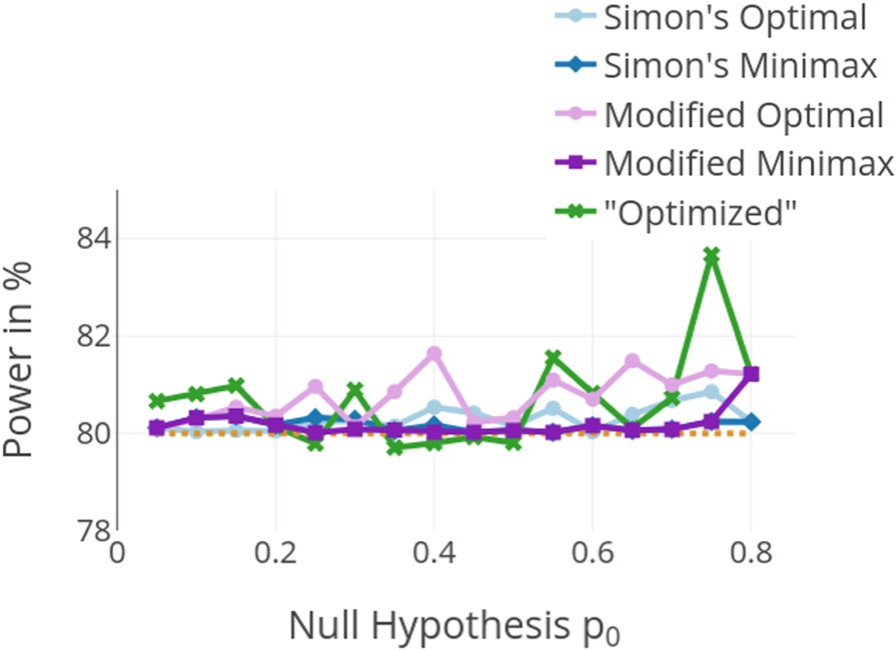
Power for $p_a-p_0 = 0.15$, $\alpha = 0.05$ and $\beta = 0.2$. Only on a few occasions, a power loss below 80% can be observed. This loss is always within the boundaries of the predefined admissible power loss $Pow_{loss}$

Figure 5 shows that the admissible power loss $Pow_{loss}$ is often not exhausted in the introduced design and often even higher power than the targeted power $1-\beta$ is reached. The targeted power $1-\beta$ is marked by a dotted line in Fig. 5. Possibly reaching a higher power than the target value is caused by the nature of binary endpoints. Sometimes the power in the introduced design is even higher than in the other four designs. Whenever the power is lower, it is within the bounds of the predefined power loss. Naturally, it is difficult to compare a design that allows power loss below the targeted value $1-\beta$ to designs without this kind of power loss. One could increase the targeted power for the design by the accepted power loss (i.e. setting $1-\beta + Pow_{loss}$ as the targeted power value in the original design without interim stopping) to get only power values higher than $1-\beta$ in the introduced optimized design.

**Fig. 6:**
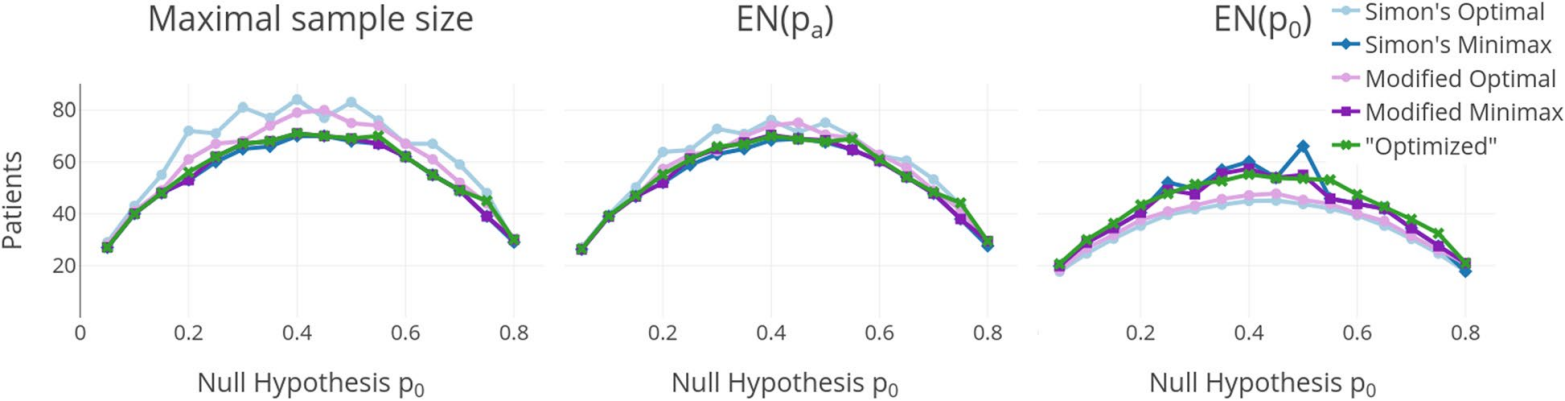
Sample sizes for $p_a-p_0 = 0.15$, $\alpha = 0.05$ and $\beta = 0.2$. Left is the maximal sample size, in the middle is the expected sample size under the alternative hypothesis, and right is the expected sample size under the null hypothesis

Figure 6 shows the maximal sample size $n=n_{max}$ and the expected sample sizes under the alternative and the null hypothesis EN($p_a$) and EN($p_0$). Simon’s optimal design and Kim’s modified version of Simon’s optimal design yield a smaller expected sample size under the null hypothesis than the introduced optimized design and the minimax designs of Simon and Kim. This was expected as a minimal expected sample size under the null hypothesis is the optimization criterion for those designs. However, they have a higher maximal sample size and expected sample size under the alternative hypothesis compared to the other designs. The optimized design’s maximal sample size and the expected sample size under the alternative hypothesis are within a close range of those from Simon’s minimax design. Only three times the difference for these measures is 3 or more patients and they are often the same as those for Kim’s modified Simon’s minimax design. Only three times the maximum sample sizes of the introduced optimized design and Kim’s modified minimax design differ from each other. Also for the expected sample size under the null hypothesis, we get quite similar values between these three designs. So if one wants to decide between these three designs, the decision can be based on the other operating characteristics instead.

The exact designs and operating characteristics for these scenarios are provided as a table in the Supplementary material. It also includes tables for the design specifications $(\alpha , \beta ) = (0.1, 0.1)$, and $(\alpha , \beta ) = (0.05, 0.1)$, as well as for other values of $Pow_{loss}$, and $\pi _{wrong}$. These additional scenarios in general give a similar picture for the comparison of the five designs. The selection of stricter boundaries for $Pow_{loss}$ and $\pi _{wrong}$ in the introduced optimized design affects only $r_1$ and $n_1$ but not *r* and *n*. It yields higher values for EN($p_0$) and EN($p_a$) and smaller values for PET($p_0$).

## Discussion

Since ensuring the correctness of interim decisions is crucial, it is necessary to investigate the implications of different futility boundaries. In the case of single-arm studies with binary endpoints, often the futility boundaries from Simon’s designs are used. However, these designs have limitations and do not yield the most promising performance characteristics, as shown in the Results section. Several authors have addressed the shortcomings of Simon’s optimal and minimax designs in the past by altering and improving the designs [10, 11, 20–22]. Kim’s modified Simon’s designs address two of the known issues of Simon’s designs - namely, that they can evaluate a high proportion of the planned sample size at the time point of the interim analysis and that they can yield an undesirably high probability of falsely declaring futility after the first stage. Kim’s designs also perform well in terms of the other investigated operating characteristics power and expected and maximal sample size. They provide an improvement to Simon’s designs and can be used effectively in clinical practice. Kim’s modified designs follow the logic of Simon’s designs with the optimal design performing better with regard to the expected sample size and the minimax design performing better with regard to the maximum sample size. The optimized design introduced here follows a different approach. The benefit of this approach is that it is easy to communicate to clinicians through a clear discussion about the desired study characteristics a priori: The sample size *n* and the efficacy boundary expressed in responders *r* are determined by designing a fixed trial, i.e. as if no interim analysis would take place. Then, the investigators can decide what power loss they would be willing to risk, how high the probability of wrongly stopping for futility should be at most, and in what range the interim analysis should be. The introduced optimized design gives the design with the highest probability of correctly stopping for futility while meeting these constraints. We also provide a straightforward R-program for implementation. Due to the fact that we didn’t increase the critical boundaries, the boundaries could be treated as non-binding boundaries. The type I error rate would still be protected in contrast to the binding rules of Simon’s optimal and minimax designs and Kim’s modified versions of those.

However, for non-binding futility stopping rules it is unclear how to make inference on interim-look adjusted confidence intervals. Therefore, in applications where inference on simultaneous confidence intervals is desired, an approach using binding futility boundaries should be preferred [23]. For Simon’s designs e.g. the method introduced by Koyama and Chen or Zhao et al. can be applied to deduce adjusted confidence intervals [24, 25].

Kim’s optimal and minimax designs and the introduced optimized design generally perform better than Simon’s optimal and minimax designs in terms of the proportion of patients evaluated at the interim analysis and the probability of wrongly stopping for futility. They should therefore be preferred over Simon’s designs and we will focus the following discussion on the selection process between the introduced optimized design and Kim’s modified designs. As the first step in this selection process, investigators need to weigh whether the expected sample size under the null hypothesis is more important to them or the proportion of correct interim decisions and the flexibility of non-binding futility stopping rules. When prioritizing the expected sample size under the null hypothesis over all else, Kim’s modified optimal design should be used. This design yields an expected sample size under the null hypothesis which is nearly as low as the one of Simon’s optimal design and it is much lower than the ones for the introduced optimized design and Kim’s minimax design. However, the maximum sample size is higher than for the other two designs, and the type I error can get quite inflated when ignoring the futility rule in the interim analysis. Therefore, one should select another design when the expected sample size under the null hypothesis is not the most important characteristic. The costs of Kim’s modified minimax design and the introduced optimized design in terms of expected and maximum sample sizes are very similar as seen in the Performance comparison section. The probability of wrongly stopping at the interim analysis is also quite close for both designs, with sometimes one, sometimes the other performing slightly better. The probability of correctly stopping for futility shows a slight tendency toward the introduced optimized design as it is often higher for this design. The power also doesn’t ground a clear decision. So when deciding between those two designs, the primary consideration is having the option for non-binding futility rules. This has a major impact on the trial. The extension of the CONSORT statement for adaptive designs [26] emphasizes the importance of stating whether binding or non-binding futility rules are used, as ignoring binding futility rules can substantially inflate the type I error rate. In the pharmaceutical industry, non-binding futility rules are typically used because the decision to continue or not is often based on more than just the number of responses. The additional flexibility of non-binding rules allows for quick adaptation to unforeseen trends and is therefore important for industry [17]. The Center for Biologics Evaluation and Research (CBER) at the FDA reported multiple cases in which supposedly binding futility boundaries were ignored. Sponsors should therefore also investigate the type I error without accounting for any futility analyses [27]. The FDA guidance document on adaptive designs notes that non-binding futility rules are often appropriate and preferred by some data monitoring committees [28]. Therefore, the introduced optimized design is recommended in this case, because it adds flexibility to futility rules for binary endpoints.

The provided R-program enables investigators to personalize the design for desired scenarios with varying values for the power loss and the probability of wrongly stopping for futility to find the most suitable parameters for the planned study. The design implications are thus easy to calculate and communicate which is a requirement of the regulatory guidance provided by the FDA [28] and EMA [29] for confirmatory trials.

## Conclusions

The choice of futility boundaries and the timing of the interim analysis can significantly impact performance characteristics. Therefore, they should be thoroughly investigated. While Simon’s designs are well-known, they may not always produce desirable operating characteristics. They may have a high proportion of incorrect interim decisions or patients evaluated at the interim analysis. Other designs incorporating futility stopping for binary endpoints should thus be preferred. For instance, Kim’s modified Simon’s designs offer a good alternative and can be recommended in case binding stopping rules are sufficient for the application. The concept introduced here that selects an optimized futility boundary also addresses these issues of Simon’s designs. It allows controlling the power loss and the probability of wrongly stopping for futility while maximizing the probability of correctly stopping for futility. Additionally, it provides greater flexibility by allowing for non-binding futility stopping rules. The costs of the introduced design compared to the minimax designs of Simon and Kim are minimal. So we suggest selecting the introduced optimized design offering higher flexibility when deciding between the three. If the expected sample size under the null hypothesis is of the highest importance for the design and binding stopping rules are acceptable Kim’s optimal design should be preferred. When selecting the introduced optimized design, we recommend additionally investigating the optimized futility boundaries over a range of possible parameters and comparing the corresponding performance characteristics in order to select the best settings for the specific use case.

### Supplementary Information

**Supplementary Material 1.**

## Data Availability

The R-source code is available on GitHub. https://github.com/MariaFrg/optimizedFutilityBinaryEndpoints.
