# Supplementary material for: Optimal futility stopping boundaries for binary endpoints
Source: BMC Med Res Methodol. 2024 Mar 28;24:80. doi: 10.1186/s12874-024-02190-w (PMC11331636; doi:10.1186/s12874-024-02190-w)
Supplement: Supplementary file 1 — Supplementary Material 1. [file 12874_2024_2190_MOESM1_ESM.zip › SupplementaryMaterial_OptimalFutilityStoppingBoundariesForBinaryEndpoints.pdf]

Additional Files for Optimal Futility Stopping Boundaries for Binary Endpoints

In this supplementary material we provide tables for a more precise comparison between Simon's optimal and minimax designs, Kim's modified versions of those and the introduced optimized design. Table 1 shows the design specifications and operating characteristics for an assumed effect of $p_a - p_0 = 0.15$ and error rates $\alpha = 0.05$ and $\beta = 0.2$ with $Pow_{loss} = 0.05$ and $\pi_{wrong} = 0.05$. Table 2 shows those for error rates $\alpha = 0.1$ and $\beta = 0.1$ with $Pow_{loss} = \pi_{wrong} = 0.05$, as well as $Pow_{loss} = \pi_{wrong} = 0.01$ and table 3 the same for $\alpha = 0.05$ and $\beta = 0.1$.

Table 1: Design specifications and operating characteristics for Simon's optimal and minimax designs, Kim's modified versions of those and the introduced optimized design with $Pow_{loss} = 0.05$ and $\pi_{wrong} = 0.05$. All designs have an assumed effect of $p_a - p_0 = 0.15$ and error rates $\alpha = 0.05$ and $\beta = 0.2$

p_0	p_a	Design method	r_1	n_1	r	n	n_1/n	PET(p_a)	PET(p_0)	$\alpha^d_{\text{no stop}}$	Power	EN(p_a)	EN(p_0)
0.05	0.2	optimized design with:											
		π_{wrong}=Pow_{loss}=0.05	0	14	3	27	0.5185	0.0440	0.4877	0.0437	0.8066	26.4283	20.6602
		Simon's optimal	0	10	3	29	0.3448	0.1074	0.5987	0.0548	0.8011	26.9599	17.6240
		Simon's minimax	0	13	3	27	0.4815	0.0550	0.5133	0.0437	0.8011	26.2303	19.8132
		Kim's optimal	0	11	3	28	0.3929	0.0859	0.5688	0.0491	0.8011	26.5397	18.3304
		Kim's minimax	0	13	3	27	0.4815	0.0550	0.5133	0.0437	0.8011	26.2303	19.8132
0.1	0.25	optimized design with:											
		π_{wrong}=Pow_{loss}=0.05	2	23	7	40	0.5750	0.0492	0.5920	0.0419	0.8081	39.1635	29.9367
		Simon's optimal	2	18	7	43	0.4186	0.1353	0.7338	0.0607	0.8003	39.6174	24.6551
		Simon's minimax	2	22	7	40	0.5500	0.0606	0.6200	0.0419	0.8032	38.9083	28.8393
		Kim's optimal	1	15	7	41	0.3659	0.0802	0.5490	0.0477	0.8029	38.9153	26.7249
		Kim's minimax	2	22	7	40	0.5500	0.0606	0.6200	0.0419	0.8032	38.9083	28.8393
0.15	0.3	optimized design with:											
		π_{wrong}=Pow_{loss}=0.05	4	28	11	48	0.5833	0.0474	0.5869	0.0478	0.8098	47.0515	36.2611
		Simon's optimal	3	19	12	55	0.3455	0.1332	0.6841	0.0603	0.8006	50.2058	30.3706
		Simon's minimax	3	23	11	48	0.4792	0.0538	0.5396	0.0478	0.8035	46.6539	34.5093
		Kim's optimal	3	21	11	49	0.4286	0.0856	0.6113	0.0550	0.8054	46.6030	31.8836
		Kim's minimax	3	23	11	48	0.4792	0.0538	0.5396	0.0478	0.8035	46.6539	34.5093
0.2	0.35	optimized design with:											
		π_{wrong}=Pow_{loss}=0.05	7	35	16	56	0.6250	0.0419	0.5993	0.0432	0.8014	55.1193	43.4140
		Simon's optimal	5	22	19	72	0.3056	0.1629	0.7326	0.0704	0.8005	63.8552	35.3681
		Simon's minimax	6	31	15	53	0.5849	0.0462	0.5711	0.0512	0.8017	51.9833	40.4363
		Kim's optimal	4	21	17	61	0.3443	0.0924	0.5860	0.0496	0.8035	57.3056	37.5597
		Kim's minimax	6	31	15	53	0.5849	0.0462	0.5711	0.0512	0.8017	51.9833	40.4363
0.25	0.4	optimized design with:											
		π_{wrong}=Pow_{loss}=0.05	10	39	21	62	0.6290	0.0450	0.6200	0.0428	0.7979	60.9639	47.7401
		Simon's optimal	5	20	23	71	0.2817	0.1256	0.6172	0.0608	0.8025	64.5945	39.5242
		Simon's minimax	16	51	20	60	0.8500	0.1319	0.8855	0.0541	0.8032	58.8133	52.0305
		Kim's optimal	6	24	22	67	0.3582	0.0960	0.6074	0.0558	0.8097	62.8737	40.8813
		Kim's minimax	10	40	21	62	0.6452	0.0352	0.5839	0.0428	0.8001	61.2251	49.1541

p_0	p_a	Design method	r_1	n_1	r	n	n_1/n	PET(p_a)	PET(p_0)	$\alpha^d_{\text{no stop}}$	Power	EN(p_a)	EN(p_0)
0.3	0.45	optimized design with:											
		π_{wrong}=Pow_{loss}=0.05	13	42	26	67	0.6269	0.0454	0.6267	0.0466	0.8089	65.8643	51.3334
		Simon's optimal	9	27	30	81	0.3333	0.1526	0.7276	0.0686	0.8024	72.7613	41.7122
		Simon's minimax	16	46	25	65	0.7077	0.1059	0.8090	0.0548	0.8029	62.9884	49.6299
		Kim's optimal	7	24	26	68	0.3529	0.0863	0.5647	0.0558	0.8013	64.2031	43.1543
		Kim's minimax	9	31	26	67	0.4627	0.0522	0.5416	0.0466	0.8008	65.1202	47.5016
0.35	0.5	optimized design with:											
		π_{wrong}=Pow_{loss}=0.05	16	44	30	68	0.6471	0.0481	0.6410	0.0461	0.7971	66.8463	52.6164
		Simon's optimal	10	27	33	77	0.3506	0.1239	0.6698	0.0605	0.8015	70.8053	43.5104
		Simon's minimax	22	55	29	66	0.8333	0.0885	0.8215	0.0511	0.8005	65.0265	56.9638
		Kim's optimal	10	28	32	74	0.3784	0.0925	0.6160	0.0556	0.8085	69.7465	45.6624
		Kim's minimax	14	42	30	68	0.6176	0.0218	0.4808	0.0461	0.8007	67.4337	55.5003
0.4	0.55	optimized design with:											
		π_{wrong}=Pow_{loss}=0.05	19	46	35	71	0.6479	0.0431	0.6329	0.0438	0.7980	69.9215	55.1777
		Simon's optimal	11	26	40	84	0.3095	0.1350	0.6737	0.0631	0.8054	76.1700	44.9266
		Simon's minimax	28	59	34	70	0.8429	0.1507	0.9028	0.0574	0.8017	68.3424	60.0695
		Kim's optimal	12	29	38	79	0.3671	0.0992	0.6374	0.0575	0.8164	74.0392	47.1292
		Kim's minimax	5	40	35	71	0.5634	0.0196	0.4402	0.0438	0.8002	70.3931	57.3532
0.45	0.6	optimized design with:											
		π_{wrong}=Pow_{loss}=0.05	21	45	38	70	0.6429	0.0483	0.6474	0.0467	0.7992	68.7919	53.8153
		Simon's optimal	12	26	41	77	0.3377	0.1082	0.6257	0.0587	0.8042	71.4824	45.0905
		Simon's minimax	19	42	38	70	0.6000	0.0375	0.5756	0.0467	0.8002	68.9499	53.8819
		Kim's optimal	14	30	43	80	0.3750	0.0971	0.6448	0.0463	0.8022	75.1472	47.7578
		Kim's minimax	19	42	38	70	0.6000	0.0375	0.5756	0.0467	0.8002	68.9499	53.8819
0.5	0.65	optimized design with:											
		π_{wrong}=Pow_{loss}=0.05	24	46	41	69	0.6667	0.0496	0.6706	0.0456	0.7981	67.8585	53.5755
		Simon's optimal	15	28	48	83	0.3373	0.1428	0.7142	0.0619	0.8015	75.1487	43.7187
		Simon's minimax	39	66	40	68	0.9706	0.1893	0.9456	0.0571	0.8013	67.6213	66.1089
		Kim's optimal	15	29	44	75	0.3867	0.0978	0.6445	0.0527	0.8032	70.5017	45.3546
		Kim's minimax	20	41	41	69	0.5942	0.0239	0.5000	0.0456	0.8006	68.3298	55.0000
0.55	0.7	optimized design with:											
		π_{wrong}=Pow_{loss}=0.05	25	44	45	70	0.6286	0.0437	0.6513	0.0452	0.8155	68.8634	53.0650
		Simon's optimal	15	26	48	76	0.3421	0.1253	0.6796	0.0602	0.8051	69.7355	42.0205
		Simon's minimax	20	35	43	67	0.5224	0.0731	0.6624	0.0501	0.8002	64.6618	45.8022
		Kim's optimal	14	25	47	74	0.3378	0.0978	0.6157	0.0550	0.8109	69.2078	43.8287
		Kim's minimax	20	35	43	67	0.5224	0.0731	0.6624	0.0501	0.8002	64.6618	45.8022
0.6	0.75	optimized design with:											
		π_{wrong}=Pow_{loss}=0.05	24	39	43	62	0.6290	0.0439	0.6365	0.0492	0.8083	60.9911	47.3599
		Simon's optimal	17	27	46	67	0.4030	0.1133	0.6913	0.0563	0.8003	62.4698	39.3490
		Simon's minimax	18	30	43	62	0.4839	0.0507	0.5689	0.0492	0.8016	60.3789	43.7949
		Kim's optimal	14	23	46	67	0.3433	0.0963	0.6116	0.0563	0.8070	62.7618	40.0877
		Kim's minimax	18	30	43	62	0.4839	0.0507	0.5689	0.0492	0.8016	60.3789	43.7949
0.65	0.8	optimized design with:											
		π_{wrong}=Pow_{loss}=0.05	24	36	41	55	0.6545	0.0424	0.6437	0.0489	0.8010	54.1939	42.7699
		Simon's optimal	12	18	49	67	0.2687	0.1329	0.6450	0.0612	0.8039	60.4871	35.3948
		Simon's minimax	20	31	41	55	0.5636	0.0327	0.5448	0.0489	0.8006	54.2149	41.9244
		Kim's optimal	16	24	45	61	0.3934	0.0892	0.6425	0.0553	0.8149	57.7007	37.2281
		Kim's minimax	20	31	41	55	0.5636	0.0327	0.5448	0.0489	0.8006	54.2149	41.9244

p_0	p_a	Design method	r_1	n_1	r	n	n_1/n	PET(p_a)	PET(p_0)	$\alpha^d_{\text{no stop}}$	Power	EN(p_a)	EN(p_0)
0.7	0.85	optimized design with:											
		π_{wrong}=Pow_{loss}=0.05	23	32	39	49	0.6531	0.0413	0.6560	0.0480	0.8073	48.2979	37.8476
		Simon's optimal	14	19	46	59	0.3220	0.1444	0.7178	0.0661	0.8067	53.2223	30.2889
		Simon's minimax	16	23	39	49	0.4694	0.0463	0.5601	0.0480	0.8008	47.7960	34.4386
		Kim's optimal	16	22	41	52	0.4231	0.0999	0.6866	0.0572	0.8099	49.0016	31.4024
		Kim's minimax	16	23	39	49	0.4694	0.0463	0.5601	0.0480	0.8008	47.7960	34.4386
0.75	0.9	optimized design with:											
		π_{wrong}=Pow_{loss}=0.05	21	27	38	45	0.6000	0.0471	0.7011	0.0446	0.8366	44.1530	32.3811
		Simon's optimal	10	13	40	48	0.2708	0.1339	0.6674	0.0611	0.8086	43.3141	24.6411
		Simon's minimax	17	22	33	39	0.5641	0.0621	0.6765	0.0512	0.8024	37.9437	27.4993
		Kim's optimal	14	18	37	44	0.4091	0.0982	0.6943	0.0523	0.8128	41.4469	25.9479
		Kim's minimax	17	22	33	39	0.5641	0.0621	0.6765	0.0512	0.8024	37.9437	27.4993
0.8	0.95	optimized design with:											
		π_{wrong}=Pow_{loss}=0.05	13	16	27	30	0.5333	0.0429	0.6482	0.0442	0.8122	29.3989	20.9258
		Simon's optimal	7	9	26	29	0.3103	0.0712	0.5638	0.0520	0.8024	27.5758	17.7242
		Simon's minimax	7	9	26	29	0.3103	0.0712	0.5638	0.0520	0.8024	27.5758	17.7242
		Kim's optimal	13	16	27	30	0.5333	0.0429	0.6482	0.0442	0.8122	29.3989	20.9258
		Kim's minimax	13	16	27	30	0.5333	0.0429	0.6482	0.0442	0.8122	29.3989	20.9258

Table 2: Design specifications and operating characteristics for Simon's optimal and minimax designs, Kim's modified versions of those and the introduced optimized design with $Pow_{loss} = 0.05$ and $\pi_{wrong} = 0.05$, as well as $Pow_{loss} = 0.01$ and $\pi_{wrong} = 0.01$. All designs have an assumed effect of $p_a - p_0 = 0.15$ and error rates $\alpha = 0.1$ and $\beta = 0.1$

p_0	p_a	Design method	r_1	n_1	r	n	n_1/n	PET(p_a)	PET(p_0)	$\alpha^d_{\text{no stop}}$	Power	EN(p_a)	EN(p_0)
0.05	0.2	optimized design with:											
		π_{wrong}=Pow_{loss}=0.05	0	14	3	32	0.4375	0.0440	0.4877	0.0738	0.8850	31.2084	23.2219
		π_{wrong}=Pow_{loss}=0.01	0	20	3	32	0.6563	0.0092	0.3406	0.0738	0.9054	31.8985	28.2538
		Simon's optimal	0	12	3	37	0.3243	0.0687	0.5404	0.1119	0.9024	35.2820	23.4910
		Simon's minimax	0	18	3	32	0.5625	0.0180	0.3972	0.0738	0.9015	31.7478	26.4390
		Kim's optimal	1	19	3	38	0.5000	0.0829	0.7547	0.1204	0.9012	36.4255	23.6606
		Kim's minimax	0	18	3	32	0.5625	0.0180	0.3972	0.0738	0.9015	31,7478	26,4390
0.1	0.25	optimized design with:											
		π_{wrong}=Pow_{loss}=0.05	2	23	6	40	0.5750	0.0492	0.5920	0.0995	0.8852	39.1635	29.9367
		π_{wrong}=Pow_{loss}=0.01	1	24	6	40	0.6000	0.0090	0.2925	0.0995	0.9022	39.8555	35.3204
		Simon's optimal	2	21	7	50	0.4200	0.0745	0.6484	0.1221	0.9008	47.8388	31.1961
		Simon's minimax	2	27	6	40	0.6750	0.0207	0.4846	0.0995	0.9001	39.7304	33.7004
		Kim's optimal	2	21	7	50	0.4200	0.0745	0.6484	0.1221	0.9008	47.8388	31.1961
		Kim's minimax	1	23	6	40	0.5750	0.0116	0.3151	0.0995	0.9012	39.8029	34.6428
0.15	0.3	optimized design with:											
		π_{wrong}=Pow_{loss}=0.05	5	33	11	53	0.6226	0.0414	0.6259	0.0907	0.8962	52.1722	40.4827
		π_{wrong}=Pow_{loss}=0.01	4	35	11	53	0.6604	0.0091	0.3807	0.0907	0.9084	52.8359	46.1465
		Simon's optimal	3	23	11	55	0.4182	0.0538	0.5396	0.1129	0.9007	53.2770	37.7319
		Simon's minimax	5	34	11	53	0.6415	0.0334	0.5973	0.0907	0.9004	52.3648	41.6510
		Kim's optimal	3	23	11	55	0.4182	0.0538	0.5396	0.1129	0.9007	53.2770	37.7319
0.2	0.35	optimized design with:											
		π_{wrong}=Pow_{loss}=0.05	8	38	16	61	0.6230	0.0474	0.6553	0.0879	0.8901	59.9109	45.9276
		π_{wrong}=Pow_{loss}=0.01	6	37	16	61	0.6066	0.0100	0.3698	0.0879	0.9040	60.7606	52.1241
		Simon's optimal	5	27	16	63	0.4286	0.0507	0.5387	0.1120	0.9019	61.1756	43.6084
		Simon's minimax	6	33	15	58	0.5690	0.0283	0.5004	0.1031	0.9003	57.2930	45.4896
		Kim's optimal	5	27	16	63	0.4286	0.0507	0.5387	0.1120	0.9019	61.1756	43.6084
		Kim's minimax	6	33	15	58	0.5690	0.0283	0.5004	0.1031	0.9003	57.2930	45.4896
0.25	0.4	optimized design with:											
		π_{wrong}=Pow_{loss}=0.05	11	42	20	64	0.6563	0.0449	0.6487	0.0993	0.8921	63.0113	49.7285
		π_{wrong}=Pow_{loss}=0.01	9	42	20	64	0.6563	0.0088	0.3711	0.0993	0.9039	63.8054	55.8369
		Simon's optimal	7	29	22	72	0.4028	0.0570	0.5568	0.1121	0.9011	69.5495	48.0588
		Simon's minimax	9	39	20	64	0.6094	0.0205	0.4756	0.0993	0.9004	63.4879	52.1092
		Kim's optimal	7	29	22	72	0.4028	0.0570	0.5568	0.1121	0.9011	69.5495	48.0588
		Kim's minimax	9	39	20	64	0.6094	0.0205	0.4756	0.0993	0.9004	63.4879	52.1092
0.3	0.45	optimized design with:											
		π_{wrong}=Pow_{loss}=0.05	15	47	26	71	0.6620	0.0474	0.6780	0.0909	0.8907	69.8629	54.7280
		π_{wrong}=Pow_{loss}=0.01	12	45	26	71	0.6338	0.0090	0.3802	0.0909	0.9030	70.7664	61.1147
		Simon's optimal	9	30	29	82	0.3659	0.0694	0.5888	0.1199	0.9005	78.3908	51.3819
		Simon's minimax	16	50	25	69	0.7246	0.0427	0.6839	0.1052	0.9016	68.1896	56.0063
		Kim's optimal	9	30	29	82	0.3659	0.0694	0.5888	0.1199	0.9005	78.3908	51.3819
		Kim's minimax	13	45	26	71	0.6338	0.0201	0.5088	0.0909	0.9003	70.4769	57.7724

p_0	p_a	Design method	r_1	n_1	r	n	n_1/n	PET(p_a)	PET(p_0)	$\alpha^d_{\text{no stop}}$	Power	EN(p_a)	EN(p_0)
0.35	0.5	optimized design with:											
		π_{wrong}=Pow_{loss}=0.05	16	44	30	72	0.6111	0.0481	0.6410	0.0964	0.8875	70.6540	54.0525
		π_{wrong}=Pow_{loss}=0.01	15	47	30	72	0.6528	0.0093	0.3913	0.0964	0.9020	71.7672	62.2163
		Simon's optimal	12	34	33	81	0.4198	0.0607	0.5919	0.1159	0.9018	78.1459	53.1787
		Simon's minimax	14	43	30	72	0.5972	0.0158	0.4365	0.0964	0.9000	71.5427	59.3407
		Kim's optimal	12	34	33	81	0.4198	0.0607	0.5919	0.1159	0.9018	78.1459	53.1787
		Kim's minimax	14	43	30	72	0.5972	0.0158	0.4365	0.0964	0.9000	71.5427	59.3407
0.4	0.55	optimized design with:											
		π_{wrong}=Pow_{loss}=0.05	21	50	35	75	0.6667	0.0444	0.6701	0.0981	0.8967	73.8905	58.2465
		π_{wrong}=Pow_{loss}=0.01	18	49	35	75	0.6533	0.0077	0.3777	0.0981	0.9081	74.8000	65.1787
		Simon's optimal	16	38	40	88	0.4318	0.0760	0.6696	0.1248	0.9000	84.1985	54.5207
		Simon's minimax	18	45	34	73	0.6164	0.0308	0.5643	0.1034	0.9001	72.1371	57.1998
		Kim's optimal	16	38	40	88	0.4318	0.0760	0.6696	0.1248	0.9000	84.1985	54.5207
		Kim's minimax	18	45	34	73	0.6164	0.0308	0.5643	0.1034	0.9001	72.1371	57.1998
0.45	0.6	optimized design with:											
		π_{wrong}=Pow_{loss}=0.05	23	49	39	75	0.6533	0.0439	0.6627	0.0913	0.8907	73.8597	57.7706
		π_{wrong}=Pow_{loss}=0.01	21	50	39	75	0.6667	0.0076	0.3900	0.0913	0.9015	74.8096	65.2509
		Simon's optimal	14	32	40	78	0.4103	0.0463	0.5165	0.1098	0.9010	75.8716	54.2399
		Simon's minimax	34	67	38	74	0.9054	0.0785	0.8572	0.1124	0.9022	73.4503	67.9996
		Kim's optimal	14	32	40	78	0.4103	0.0463	0.5165	0.1098	0.9010	75.8716	54.2399
		Kim's minimax	22	50	39	75	0.6667	0.0160	0.5019	0.0913	0.9002	74.5991	62.4523
0.5	0.65	optimized design with:											
		π_{wrong}=Pow_{loss}=0.05	24	46	41	72	0.6389	0.0496	0.6706	0.0973	0.8891	70.7096	54.5636
		π_{wrong}=Pow_{loss}=0.01	22	47	41	72	0.6528	0.0080	0.3854	0.0973	0.9031	71.8005	62.3642
		Simon's optimal	18	35	47	84	0.4167	0.0682	0.6321	0.1149	0.9004	80.6594	53.0290
		Simon's minimax	19	40	41	72	0.5556	0.0173	0.4373	0.0973	0.9001	71.4470	58.0059
		Kim's optimal	18	35	47	84	0.4167	0.0682	0.6321	0.1149	0.9004	80.6594	53.0290
		Kim's minimax	19	40	41	72	0.5556	0.0173	0.4373	0.0973	0.9001	71.4470	58.0059
0.55	0.7	optimized design with:											
		π_{wrong}=Pow_{loss}=0.05	27	47	44	71	0.6620	0.0458	0.6841	0.0961	0.8967	69.8997	54.5807
		π_{wrong}=Pow_{loss}=0.01	23	44	44	71	0.6197	0.0100	0.4143	0.0961	0.9081	70.7302	59.8149
		Simon's optimal	19	34	46	75	0.4533	0.0571	0.6063	0.1110	0.9001	72.6576	50.1410
		Simon's minimax	35	58	43	70	0.8286	0.0744	0.8288	0.1144	0.9006	69.1071	60.0540
		Kim's optimal	19	34	46	75	0.4533	0.0571	0.6063	0.1110	0.9001	72.6576	50.1410
		Kim's minimax	16	31	44	71	0.4366	0.0239	0.4192	0.0961	0.9003	70.0451	54.2311
0.6	0.75	optimized design with:											
		π_{wrong}=Pow_{loss}=0.05	26	42	43	64	0.6563	0.0416	0.6554	0.0953	0.8915	63.0842	49.5818
		π_{wrong}=Pow_{loss}=0.01	24	42	43	64	0.6563	0.0086	0.4090	0.0953	0.9004	63.8110	55.0021
		Simon's optimal	21	34	47	71	0.4789	0.0610	0.6458	0.1168	0.9036	68.7431	47.1043
		Simon's minimax	25	43	43	64	0.6719	0.0114	0.4587	0.0953	0.9002	63.7610	54.3663
		Kim's optimal	21	34	47	71	0.4789	0.0610	0.6458	0.1168	0.9036	68.7431	47.1043
		Kim's minimax	22	39	43	64	0.6094	0.0086	0.3807	0.0953	0.9001	63.7844	54.4824
0.65	0.8	optimized design with:											
		π_{wrong}=Pow_{loss}=0.05	27	40	44	61	0.6557	0.0432	0.6857	0.0945	0.9009	60.0919	46.6006
		π_{wrong}=Pow_{loss}=0.01	25	40	44	61	0.6557	0.0079	0.4279	0.0945	0.9118	60.8338	52.0137
		Simon's optimal	20	30	45	63	0.4762	0.0611	0.6425	0.1133	0.9000	60.9841	41.7988
		Simon's minimax	22	33	43	60	0.5500	0.0508	0.6430	0.1101	0.9012	58.6274	42.6400
		Kim's optimal	20	30	45	63	0.4762	0.0611	0.6425	0.1133	0.9000	60.9841	41.7988
		Kim's minimax	22	33	43	60	0.5500	0.0508	0.6430	0.1101	0.9012	58.6274	42.6400

p_0	p_a	Design method	r_1	n_1	r	n	n_1/n	PET(p_a)	PET(p_0)	$\alpha^d_{\text{no stop}}$	Power	EN(p_a)	EN(p_0)
0.7	0.85	optimized design with:											
		π_{wrong}=Pow_{loss}=0.05	24	33	41	53	0.6226	0.0495	0.6957	0.0906	0.8948	52.0104	39.0863
		π_{wrong}=Pow_{loss}=0.01	23	34	41	53	0.6415	0.0087	0.4455	0.0906	0.9091	52.8350	44.5347
		Simon's optimal	14	20	45	59	0.3390	0.0673	0.5836	0.1145	0.9010	56.3750	36.2385
		Simon's minimax	15	22	40	52	0.4231	0.0368	0.5058	0.1049	0.9029	50.8948	36.8253
		Kim's optimal	14	20	45	59	0.3390	0.0673	0.5836	0.1145	0.9010	56.3750	36.2385
		Kim's minimax	15	22	40	52	0.4231	0.0368	0.5058	0.1049	0.9029	50.8948	36.8253
0.75	0.9	optimized design with:											
		π_{wrong}=Pow_{loss}=0.05	20	26	33	40	0.6500	0.0399	0.6629	0.0962	0.8941	39.4420	30.7200
		π_{wrong}=Pow_{loss}=0.01	18	25	33	40	0.6250	0.0095	0.4389	0.0962	0.9005	39.8579	33.4165
		Simon's optimal	12	16	39	48	0.3333	0.0684	0.5950	0.1190	0.9039	45.8110	28.9596
		Simon's minimax	20	27	33	40	0.6750	0.0147	0.5292	0.0962	0.9005	39.8093	33.1208
		Kim's optimal	12	16	39	48	0.3333	0.0684	0.5950	0.1190	0.9039	45.8110	28.9596
		Kim's minimax	19	26	33	40	0.6500	0.0119	0.4846	0.0962	0.9005	39.8338	33.2155
0.8	0.95	optimized design with:											
		π_{wrong}=Pow_{loss}=0.05	13	16	28	32	0.5000	0.0429	0.6482	0.0931	0.9104	31.3130	21.6295
		π_{wrong}=Pow_{loss}=0.01	13	17	28	32	0.5313	0.0088	0.4511	0.0931	0.9262	31.8680	25.2331
		Simon's optimal	5	7	27	31	0.2258	0.0444	0.4233	0.1070	0.9050	29.9349	20.8412
		Simon's minimax	5	7	27	31	0.2258	0.0444	0.4233	0.1070	0.9050	29.9349	20.8412
		Kim's optimal	13	16	27	31	0.5161	0.0429	0.6482	0.1070	0.9162	30.3559	21.2777
		Kim's minimax	13	16	27	31	0.5161	0.0429	0.6482	0.1070	0.9162	30.3559	21.2777

Table 3: Design specifications and operating characteristics for Simon's optimal and minimax designs, Kim's modified versions of those and the introduced optimized design with $Pow_{loss} = 0.05$ and $\pi_{wrong} = 0.05$, as well as $Pow_{loss} = 0.01$ and $\pi_{wrong} = 0.01$. All designs have an assumed effect of $p_a - p_0 = 0.15$ and error rates $\alpha = 0.05$ and $\beta = 0.1$

p_0	p_a	Design method	r_1	n_1	r	n	n_1/n	PET(p_a)	PET(p_0)	$\alpha^d_{\text{no stop}}$	Power	EN(p_a)	EN(p_0)
0.05	0.2	optimized design with:											
		π_{wrong}=Pow_{loss}=0.05	1	22	4	38	0.5789	0.0480	0.6982	0.0397	0.8836	37.2326	26.8296
		π_{wrong}=Pow_{loss}=0.01	0	21	4	38	0.5526	0.0092	0.3406	0.0397	0.8992	37.8432	32.2105
		Simon's optimal	1	21	4	41	0.5122	0.0576	0.7170	0.0525	0.9017	39.8471	26.6606
		Simon's minimax	1	29	4	38	0.7632	0.0128	0.5708	0.0397	0.9004	37.8851	32.8629
		Kim's optimal	1	21	4	41	0.5122	0.0576	0.7170	0.0525	0.9017	39.8471	26.6606
		Kim's minimax	0	23	4	38	0.6053	0.0059	0.3074	0.0397	0.9005	37.9115	33.3896
0.1	0.25	optimized design with:											
		π_{wrong}=Pow_{loss}=0.05	4	34	9	55	0.6182	0.0491	0.7504	0.0444	0.8935	53.9690	39.2414
		π_{wrong}=Pow_{loss}=0.01	2	31	9	55	0.5636	0.0084	0.3886	0.0444	0.9094	54.7975	45.6739
		Simon's optimal	2	21	10	66	0.3182	0.0745	0.6484	0.0621	0.9018	62.6464	36.8216
		Simon's minimax	3	31	9	55	0.5636	0.0307	0.6238	0.0444	0.9006	54.2623	40.0281
		Kim's optimal	3	28	9	57	0.4912	0.0551	0.6946	0.0548	0.9013	55.4011	36.8576
		Kim's minimax	3	31	9	55	0.5636	0.0307	0.6238	0.0444	0.9006	54.2623	40.0281
0.15	0.3	optimized design with:											
		π_{wrong}=Pow_{loss}=0.05	7	41	14	64	0.6406	0.0458	0.7335	0.0491	0.8889	62.9460	47.1285
		π_{wrong}=Pow_{loss}=0.01	5	40	14	64	0.6250	0.0086	0.4325	0.0491	0.9015	63.7932	53.6200
		Simon's optimal	5	30	17	82	0.3659	0.0766	0.7106	0.0591	0.9007	78.0171	45.0501
		Simon's minimax	6	42	14	64	0.6563	0.0155	0.5545	0.0491	0.9003	63.6592	51.8005
		Kim's optimal	5	30	17	82	0.3659	0.0766	0.7106	0.0591	0.9007	78.0171	45.0501
		Kim's minimax	6	42	14	64	0.6563	0.0155	0.5545	0.0491	0.9003	63.6592	51.8005
0.2	0.35	optimized design with:											
		π_{wrong}=Pow_{loss}=0.05	11	49	21	77	0.6364	0.0419	0.7355	0.0454	0.8931	75.8271	56.4073
		π_{wrong}=Pow_{loss}=0.01	9	49	21	77	0.6364	0.0086	0.4717	0.0454	0.9043	76.7603	63.7923
		Simon's optimal	8	37	22	83	0.4458	0.0588	0.6859	0.0568	0.9009	80.2964	51.4482
		Simon's minimax	8	42	21	77	0.5455	0.0190	0.5309	0.0454	0.9002	76.3364	58.4177
		Kim's optimal	8	37	22	83	0.4458	0.0588	0.6859	0.0568	0.9009	80.2964	51.4482
		Kim's minimax	8	42	21	77	0.5455	0.0190	0.5309	0.0454	0.9002	76.3364	58.4177
0.25	0.4	optimized design with:											
		π_{wrong}=Pow_{loss}=0.05	15	54	27	83	0.6506	0.0429	0.7402	0.0467	0.8889	81.7566	61.5336
		π_{wrong}=Pow_{loss}=0.01	13	55	27	83	0.6627	0.0081	0.4794	0.0467	0.8996	82.7720	69.5761
		Simon's optimal	10	37	31	99	0.3737	0.0722	0.6909	0.0614	0.9002	94.5250	56.1622
		Simon's minimax	13	57	27	83	0.6867	0.0048	0.4190	0.0467	0.9001	82.8751	72.1067
		Kim's optimal	10	37	31	99	0.3737	0.0722	0.6909	0.0614	0.9002	94.5250	56.1622
		Kim's minimax	11	52	27	83	0.6265	0.0032	0.3232	0.0467	0.9001	82.9001	72.9803
0.3	0.45	optimized design with:											
		π_{wrong}=Pow_{loss}=0.05	21	62	35	93	0.6667	0.0500	0.7911	0.0450	0.8930	91.4502	68.4770
		π_{wrong}=Pow_{loss}=0.01	18	61	35	93	0.6559	0.0097	0.5297	0.0450	0.9068	92.6893	76.0496
		Simon's optimal	13	40	40	110	0.3636	0.0751	0.7032	0.0614	0.9012	104.7461	60.7726
		Simon's minimax	27	77	33	88	0.8750	0.0497	0.8625	0.0516	0.9006	87.4530	78.5122
		Kim's optimal	13	40	40	110	0.3636	0.0751	0.7032	0.0614	0.9012	104.7461	60.7726
		Kim's minimax	14	46	34	91	0.5055	0.0316	0.5969	0.0521	0.9015	89.5792	64.1394

p_0	p_a	Design method	r_1	n_1	r	n	n_1/n	PET(p_a)	PET(p_0)	$\alpha^d_{\text{no stop}}$	Power	EN(p_a)	EN(p_0)
0.35	0.5	optimized design with:											
		π_{wrong}=Pow_{loss}=0.05	24	62	41	96	0.6458	0.0490	0.7737	0.0471	0.8928	94.3348	69.6938
		π_{wrong}=Pow_{loss}=0.01	22	64	41	96	0.6667	0.0084	0.5157	0.0471	0.9072	95.7303	79.4976
		Simon's optimal	16	43	44	105	0.4095	0.0631	0.6828	0.0579	0.9000	101.0850	62.6664
		Simon's minimax	16	46	40	94	0.4894	0.0270	0.5552	0.0517	0.9004	92.7022	67.3511
		Kim's optimal	16	43	44	105	0.4095	0.0631	0.6828	0.0579	0.9000	101.0850	62.6664
		Kim's minimax	16	46	40	94	0.4894	0.0270	0.5552	0.0517	0.9004	92.7022	67.3511
0.4	0.55	optimized design with:											
		π_{wrong}=Pow_{loss}=0.05	27	62	45	94	0.6596	0.0464	0.7593	0.0491	0.8884	92.5164	69.7037
		π_{wrong}=Pow_{loss}=0.01	23	59	45	94	0.6277	0.0097	0.4930	0.0491	0.8995	93.6602	76.7459
		Simon's optimal	19	45	49	104	0.4327	0.0582	0.6786	0.0578	0.9002	100.5661	63.9612
		Simon's minimax	24	62	45	94	0.6596	0.0072	0.4725	0.0491	0.9000	93.7688	78.8802
		Kim's optimal	19	45	49	104	0.4327	0.0582	0.6786	0.0578	0.9002	100.5661	63.9612
		Kim's minimax	24	62	45	94	0.6596	0.0072	0.4725	0.0491	0.9000	93.7688	78.8802
0.45	0.6	optimized design with:											
		π_{wrong}=Pow_{loss}=0.05	31	64	52	98	0.6531	0.0402	0.7518	0.0444	0.8926	96.6319	72.4385
		π_{wrong}=Pow_{loss}=0.01	29	65	52	98	0.6633	0.0086	0.5265	0.0444	0.9018	97.7146	80.6263
		Simon's optimal	19	40	60	116	0.3448	0.0744	0.6844	0.0610	0.9002	110.3493	63.9845
		Simon's minimax	49	93	50	95	0.9789	0.0919	0.9442	0.0553	0.9018	94.8162	93.1115
		Kim's optimal	19	40	60	116	0.3448	0.0744	0.6844	0.0610	0.9002	110.3493	63.9845
		Kim's minimax	26	58	52	98	0.5918	0.0138	0.5437	0.0444	0.9003	97.4476	76.2513
0.5	0.65	optimized design with:											
		π_{wrong}=Pow_{loss}=0.05	31	58	54	93	0.6237	0.0459	0.7441	0.0483	0.8880	91.3950	66.9572
		π_{wrong}=Pow_{loss}=0.01	30	61	54	93	0.6559	0.0080	0.5000	0.0483	0.9006	92.7437	77.0000
		Simon's optimal	22	42	60	105	0.4000	0.0623	0.6780	0.0590	0.9014	101.0739	62.2850
		Simon's minimax	28	57	54	93	0.6129	0.0099	0.5000	0.0483	0.9001	92.6424	75.0000
		Kim's optimal	22	42	60	105	0.4000	0.0623	0.6780	0.0590	0.9014	101.0739	62.2850
		Kim's minimax	28	57	54	93	0.6129	0.0099	0.5000	0.0483	0.9001	92.6424	75.0000
0.55	0.7	optimized design with:											
		π_{wrong}=Pow_{loss}=0.05	36	61	58	92	0.6630	0.0443	0.7755	0.0480	0.8969	90.6255	67.9610
		π_{wrong}=Pow_{loss}=0.01	33	60	58	92	0.6522	0.0100	0.5498	0.0480	0.9078	91.6813	74.4048
		Simon's optimal	22	38	68	110	0.3455	0.0762	0.6974	0.0619	0.9005	104.5146	59.7903
		Simon's minimax	50	81	56	89	0.9101	0.0686	0.9087	0.0529	0.9009	88.4512	81.7303
		Kim's optimal	22	38	68	110	0.3455	0.0762	0.6974	0.0619	0.9005	104.5146	59.7903
		Kim's minimax	26	47	58	92	0.5109	0.0235	0.5736	0.0480	0.9016	90.9436	66.1891
0.6	0.75	optimized design with:											
		π_{wrong}=Pow_{loss}=0.05	36	56	58	85	0.6588	0.0486	0.7844	0.0468	0.8908	83.5910	62.2525
		π_{wrong}=Pow_{loss}=0.01	32	54	58	85	0.6353	0.0080	0.5073	0.0468	0.9032	84.7530	69.2725
		Simon's optimal	21	34	64	95	0.3579	0.0610	0.6458	0.0566	0.9012	91.2792	55.6044
		Simon's minimax	48	72	57	84	0.8571	0.0703	0.8999	0.0553	0.9003	83.1566	73.2007
		Kim's optimal	21	34	64	95	0.3579	0.0610	0.6458	0.0566	0.9012	91.2792	55.6044
		Kim's minimax	29	48	58	85	0.5647	0.0184	0.5778	0.0468	0.9001	84.3209	63.6200
0.65	0.8	optimized design with:											
		π_{wrong}=Pow_{loss}=0.05	33	48	55	75	0.6400	0.0437	0.7541	0.0485	0.8898	73.8192	54.6396
		π_{wrong}=Pow_{loss}=0.01	31	48	55	75	0.6400	0.0093	0.5301	0.0485	0.8998	74.7484	60.6882
		Simon's optimal	21	31	67	93	0.3333	0.0746	0.6890	0.0605	0.9016	88.3748	50.2848
		Simon's minimax	34	52	55	75	0.6933	0.0099	0.5750	0.0485	0.9000	74.7732	61.7753
		Kim's optimal	21	31	67	93	0.3333	0.0746	0.6890	0.0605	0.9016	88.3748	50.2848
		Kim's minimax	29	46	55	75	0.6133	0.0058	0.4448	0.0485	0.9001	74.8330	62.1004

p_0	p_a	Design method	r_1	n_1	r	n	n_1/n	PET(p_a)	PET(p_0)	$\alpha^d_{\text{no stop}}$	Power	EN(p_a)	EN(p_0)
0.7	0.85	optimized design with:											
		π_{wrong}=Pow_{loss}=0.05	32	43	54	69	0.6232	0.0488	0.7852	0.0480	0.8992	67.7306	48.5842
		π_{wrong}=Pow_{loss}=0.01	30	43	54	69	0.6232	0.0085	0.5441	0.0480	0.9142	68.7794	54.8540
		Simon's optimal	18	25	61	79	0.3165	0.0695	0.6593	0.0608	0.9041	75.2455	43.3954
		Simon's minimax	33	44	53	68	0.6471	0.0566	0.8115	0.0556	0.9023	66.6417	48.5244
		Kim's optimal	23	31	63	82	0.3780	0.0822	0.7552	0.0678	0.9015	77.8076	43.4859
		Kim's minimax	33	44	53	68	0.6471	0.0566	0.8115	0.0556	0.9023	66.6417	48.5244
0.75	0.9	optimized design with:											
		π_{wrong}=Pow_{loss}=0.05	27	34	46	55	0.6182	0.0481	0.7820	0.0454	0.8929	53.9890	38.5788
		π_{wrong}=Pow_{loss}=0.01	27	36	46	55	0.6545	0.0077	0.5637	0.0454	0.9056	54.8542	44.2897
		Simon's optimal	18	23	52	63	0.3651	0.0731	0.7168	0.0584	0.9004	60.0755	34.3285
		Simon's minimax	19	25	45	54	0.4630	0.0334	0.6217	0.0525	0.9019	53.0314	35.9701
		Kim's optimal	18	23	52	63	0.3651	0.0731	0.7168	0.0584	0.9004	60.0755	34.3285
		Kim's minimax	19	25	45	54	0.4630	0.0334	0.6217	0.0525	0.9019	53.0314	35.9701
0.8	0.95	optimized design with:											
		π_{wrong}=Pow_{loss}=0.05	24	28	39	44	0.6364	0.0491	0.8398	0.0440	0.9161	43.2148	30.5629
		π_{wrong}=Pow_{loss}=0.01	21	26	39	44	0.5909	0.0085	0.6167	0.0440	0.9325	43.8468	32.9001
		Simon's optimal	16	19	37	42	0.4524	0.0665	0.7631	0.0580	0.9031	40.4694	24.4485
		Simon's minimax	31	35	35	40	0.8750	0.0958	0.9395	0.0759	0.9003	39.5212	35.3026
		Kim's optimal	16	19	37	42	0.4524	0.0665	0.7631	0.0580	0.9031	40.4694	24.4485
		Kim's minimax	16	19	37	42	0.4524	0.0665	0.7631	0.0580	0.9031	40.4694	24.4485
